# Heart Rate Variability and MOXO d-CPT Relationship in Children with in Attention Deficit Hyperactivity Disorder

**DOI:** 10.1007/s10484-026-09766-w

**Published:** 2026-01-27

**Authors:** Sultan Tarlacı, Yaren Kaya Topal

**Affiliations:** 1 Medical Faculty, Department of Neurology and Neuroscience, Üsküdar University, İstanbul, Turkey; 2 Department of Neuroscience, Üsküdar University, İstanbul, Turkey

**Keywords:** ADHD, Continuous performance tests, CPT, MOXO d-CPT, Hearth rate variability, HRV

## Abstract

This study examined heart rate variability (HRV) in children with attention deficit hyperactivity disorder (ADHD) using a case–control design. A total of 52 children aged 6–12 years participated. ADHD diagnosis was confirmed by a child psychiatrist according to DSM-5 criteria, and comorbidities (e.g., anxiety, ODD) were excluded. Participants were then stratified into ADHD (n = 33; 17 boys, 16 girls) and control groups (n = 19; 12 boys, 7 girls), with MOXO d-CPT used to characterize performance profiles. Resting-state HRV was recorded for 5 min using the validated Polar H10 device. Time-domain (SDNN, RMSSD), frequency-domain (VLF, LF, HF), and non-linear indices were analyzed. The Good performance group demonstrated significantly lower VLF power compared to the Weak group (*η*^2^ = 0.176, *p* < 0.05), and the SNS Index was significantly higher in the Weak hyperactivity group (d = 0.49, *p* = 0.048), indicating increased sympathetic activation associated with poorer performance. Non-significant trends were observed for SDNN (*η*^2^ = 0.08) and RMSSD (*η*^2^ = 0.07), suggesting modest parasympathetic differences, though these did not reach statistical significance. These findings highlight VLF and the SNS Index as the most robust HRV metrics associated with impulsivity and hyperactivity performance on the MOXO d-CPT, while other HRV domains showed only preliminary or non-significant effects. Future longitudinal studies are needed to evaluate HRV’s potential for monitoring treatment response and its specificity to cognitive subdomains in ADHD.

## Introduction

The cortical and subcortical structures of the limbic system form the core of the brain’s autonomic regulation, integrating visceromotor, emotional, and cognitive processes within the central autonomic network (CAN). Empirical evidence highlights the contribution of cortical regions—including the anterior cingulate cortex (ACC), medial and inferior frontal gyri, anterior and posterior insula, hippocampus, ventral striatum, amygdala, and hypothalamus—to higher-order autonomic and attentional control (Benarroch, 1993; Beissner et al., 2013; Harper et al., 2000; Ruiz Vargas et al., 2016). The ventromedial prefrontal cortex (vmPFC), with its dense reciprocal connections to limbic and autonomic structures, integrates affective and motivational signals with executive regulation (Fuster, 1985, 1997; Mesulam & Mufson, 1982).

Modern neuroimaging work supports these early observations, extending them through large-scale meta-analyses and network-based models. The striatum modulates behavior through direct and indirect pathways that govern response inhibition and selection, while prefrontal regions orchestrate sustained attention and cognitive control (Beissner et al., 2013; Hécaen, 1964). Contemporary ENIGMA consortium findings demonstrate modest but consistent structural alterations in ADHD, including reduced subcortical volumes and delayed cortical maturation (Hoogman et al., 2017, 2020). Effect sizes in these regions are typically small to moderate (Hedges’ g ≈ 0.30–0.45), underscoring heterogeneity across cohorts and developmental stages. Frontal abnormalities—particularly in orbitofrontal cortex, dorsal prefrontal regions, and precentral areas—show subtle thinning or hypovolumes rather than uniform deficits (Fernández-Jaén et al., 2014; Proal et al., 2011). Lateralization patterns remain debated: while some studies report right-lateralized differences in inferior frontal gyrus and insula, effect size variability and occasional null findings caution against overgeneralization (Lopez-Larson et al., 2012; Yang et al., 2015).

The ACC plays a pivotal role in linking cognitive control with autonomic and emotional inputs, supporting effortful attention, conflict monitoring, and error detection (Mesulam et al., 2001; Posner et al., 1988). Structural alterations in the dorsal and midcingulate cortices have been associated with ADHD symptom severity, particularly perseverance and performance monitoring deficits (Bush et al., 1999, 2009; Klein et al., 2021). Electrical stimulation studies demonstrate the ACC’s capacity to evoke distinct autonomic responses—including pupillary dilation and cardiorespiratory shifts—highlighting its integrative role (Pool, 1954; Talairach et al., 1973).

The anterior insula, tightly coupled with the dorsal ACC, anchors the salience network and mediates adaptive switching between the default mode and executive control networks (Botvinick et al., 2004; Sridharan et al., 2008). Gray matter reductions and connectivity disruptions in bilateral insulae have been documented in ADHD, though lateralization patterns vary across studies (Chouchou et al., 2017; Li et al., 2015; Lopez-Larson et al., 2012; Maier et al., 2023). Functional alterations in the parahippocampal gyrus (PHG) and related limbic regions further reflect aberrant integration across attention and emotion circuits (Gehricke et al., 2017; Qian et al., 2021). These findings align with network-level models emphasizing the distributed nature of attentional dysregulation.

Recent pediatric work has emphasized links between structural variations in limbic–insular hubs and performance on cognitive tasks such as the MOXO d-CPT. For instance, severity correlates with morphology of the right triangular inferior frontal gyrus, whereas timing relates to right precentral and superior frontal areas, and hyperactivity corresponds to alterations in the left posterior insula and right PHG (Tarlacı & Çolak, 2026). These relationships reinforce the centrality of salience, control, and limbic circuits in ADHD-related behavioral regulation.

Against this neurobiological background, heart rate variability (HRV) offers a physiophysiological window into autonomic–cognitive interactions tied to these networks. The neurovisceral integration model posits that vagally mediated HRV reflects prefrontal inhibitory capacity and executive control (Thayer et al., 2009, 2012). Studies have reported associations between HRV and attention, timing, impulsivity, and regulatory behaviors in children (Althaus et al., 1998; DeGangi et al., 1991; Denver et al., 2007). Furthermore, recent research indicates HRV alterations in ADHD, although findings remain mixed and are moderated by developmental and contextual factors (Çetin & Tarlacı, 2022; Nooripour et al., 2024).

Given these neural and autonomic interdependencies, the present study aims to investigate associations between HRV parameters, weight indices, and distinct performance domains of the MOXO d-CPT—including attention, timing, impulsivity, and hyperactivity—in children with ADHD. This approach may clarify shared neurophysiological mechanisms underlying cognitive and behavioral manifestations of the disorder.

## Materials and Method

### Participants

Participants were recruited from outpatient clinics and community advertisements. ADHD diagnoses were confirmed by a child psychiatrist according to DSM-5 criteria. The control group consisted of typically developing children with no history of neurodevelopmental, psychiatric, or chronic medical conditions, as reported by parents. Inclusion criteria were: (1) age between 6 and 12 years, and (2) absence of medication use affecting heart rate or autonomic nervous system activity. Exclusion criteria included chronic illness, intellectual disability, pervasive developmental disorders, psychotic disorders, and current use of ADHD medication. After applying these criteria, 52 children were enrolled: 33 in the ADHD group (17 boys, 16 girls) and 19 in the control group (12 boys, 7 girls).

### Procedure

All families received detailed study information, and written informed consent was obtained from parents prior to participation. The study adhered to the Declaration of Helsinki. Parents completed a sociodemographic questionnaire at the start of the session. Before administration of the MOXO d-CPT, resting-state heart rate variability (HRV) was recorded using the Polar H10 sensor. Participants were seated in a quiet, temperature-controlled room, and the 5 min HRV acquisition followed standard short-term HRV guidelines. Immediately after HRV recording, participants completed the 15-min MOXO d-CPT. All assessments were conducted within a single visit, and no follow-up measurements were obtained.

### HRV Acquisition and Analysis

Heart rate variability (HRV) was measured using the Polar H10 heart rate sensor, selected for its high accuracy in detecting RR intervals compared with standard electrocardiography (Hinde, 2021; Lu, 2023). Although multiple wearable devices are available for HRV assessment, the Polar H10 has been consistently validated as one of the most precise options. A 5 min resting recording protocol was applied in a quiet, temperature-controlled room with participants in a seated position to standardize conditions. The chest-strap device transmitted data via Bluetooth, and the recorded RR intervals were processed using Kubios HRV software (Version 3.5; Kuopio, Finland).

All RR interval recordings were visually inspected to identify and remove premature beats or artifacts. Data were excluded if more than 5% of beats were classified as ectopic or artifact. Cleaned normal-to-normal (NN) interval series were then used to compute HRV parameters across time-domain, frequency-domain, and nonlinear metrics. Time-domain measures included mean RR interval (RR), mean heart rate (HR), standard deviation of NN intervals (SDNN), root mean square of successive differences (RMSSD), NN50, pNN50, RR triangular index, triangular interpolation of NN interval histogram (TINN), and the Stress Index. Frequency-domain analysis was performed using the Welch periodogram to calculate power in very low-frequency (VLF: 0.003–0.04 Hz), low-frequency (LF: 0.04–0.15 Hz), and high-frequency (HF: 0.15–0.40 Hz) bands, with the LF/HF ratio serving as an index of sympathovagal balance and HF power reflecting parasympathetic activity. Nonlinear and Poincaré-derived measures included approximate entropy (ApEn), sample entropy (SampEn), short-term variability (SD1), long-term variability (SD2), and the SD2/SD1 ratio. These comprehensive analyses provided a detailed assessment of autonomic regulation and HRV dynamics.

### MOXO d-CPT

When diagnosing ADHD, the MOXO Continuous Performance Test (MOXO d-CPT) can be utilized as a supplemental tool (Slobodin et al., 2018). The MOXO d-CPT is an objective continuous performance test that incorporates visual, auditory, and mixed auditory-visual distractors. It assesses four subscales: impulsivity, hyperactivity, timing, and attentiveness (Berger et al., 2021). Performance is further evaluated across four levels (good, standard, weak, and difficulty), with performance decreasing numerically from Level 1 to Level 4. Each subscale’s severity (attention, timing, hyperactivity, and impulsivity) is classified into four categories: low, medium, high, and extreme.

The MOXO d-CPT performance level is determined based on standardized z-scores. A z-score of 0 or higher indicates good performance (high normal range), − 0.825 to 0 represents standard performance, − 1.65 to − 0.825 indicates weak performance, and − 1.65 or lower suggests significant performance difficulty, placing the individual outside the normal range. Z-scores reflect relative performance within the assessment index. Scores above average are not explicitly recorded, as the test focuses on deviations below the norm.

Severity levels quantify the degree of performance impairment. Low severity corresponds to z-scores between−1.95 and − 1.65 (~ 2% of the population), medium severity − 2.25 to − 1.95 (~ 1.5%), high severity − 2.55 to − 2.25 (~ 0.75%), and extreme severity − 2.55 or lower (~ 0.75%). Higher severity levels indicate greater deviations from the norm.

The MOXO d-CPT was administered in a standardized environment on a desktop computer by a trained researcher. Participants were instructed to respond to target stimuli and ignore non-targets amidst visual and auditory distractors presented in each of the test’s eight levels. The test was conducted according to the manufacturer’s standardized instructions.

### Statistical Analysis

Data were analyzed using SPSS 26 and JASP 0.9. A variety of statistical approaches were used to guarantee the reliability of the findings. Data normality was assessed using the Shapiro–Wilk test to ensure parametric assumptions were met. First, the homogeneity of variances between the groups was evaluated using the Brown-Forsythe Test and Levene’s F Test. A two-way ANOVA was selected to examine the interaction between diagnosis and performance groups on HRV parameters, as this method allows for assessment of the main effects of two independent variables and their interaction. Post-hoc Tukey’s HSD tests were applied following significant ANOVA results to identify specific group differences. Student’s t-test was used for two-group comparisons (e.g., ADHD vs. Normal).

Eta-squared (*η*^2^) effect sizes were calculated for comparisons involving more than two groups in ANOVA, whereas Cohen’s d effect sizes were calculated for pairwise comparisons. Large effect sizes are generally represented by *η*^2^ values of 0.14, medium effect sizes by 0.06, and tiny effect sizes by 0.01. Effect sizes for Cohen’s d are commonly interpreted as follows: A modest effect is 0.2, a medium effect is 0.5, and a large influence is 0.8 or greater. All tests were conducted using a 95% confidence interval. A p-value of less than 0.05 was considered statistically significant.

### Ethical Approval

In accordance with the Declaration of Helsinki, written informed consent was obtained from all parents, and age-appropriate verbal assent was obtained from all child participants. The study protocol included monitoring for any signs of distress during testing, with no adverse events reported. This study was authorized by the Ethics Board of Üsküdar University in İstanbul, Turkey (61,351,342/aralık2023-29).

## Results

The ADHD group included 33 children (17 males [51.5%], 16 females [48.5%]), while the control group comprised 19 children (12 males [63.2%], 7 females [36.8%]). Medication use was reported in 2 participants (6.1%) in the ADHD group and 4 participants (21.1%) in the control group. Demographic and perinatal characteristics did not differ significantly between groups. The mean age was 8.24 ± 1.48 years in the ADHD group and 8.68 ± 1.80 years in the control group (t = −0.96, *p* = 0.343, Cohen’s d = 0.26; Levene’s F = 1.505, *p* = 0.226). Gestation time, delivery duration, and birth weight were likewise comparable (all *p* > 0.40). Overall, no statistically significant differences were observed in age, sex, gestation time, delivery time, birth weight, or medication use, confirming demographic homogeneity between groups.

### HRV Results: ADHD vs. Normal Group

#### Time-Domain Measures

**Fig. 1:**
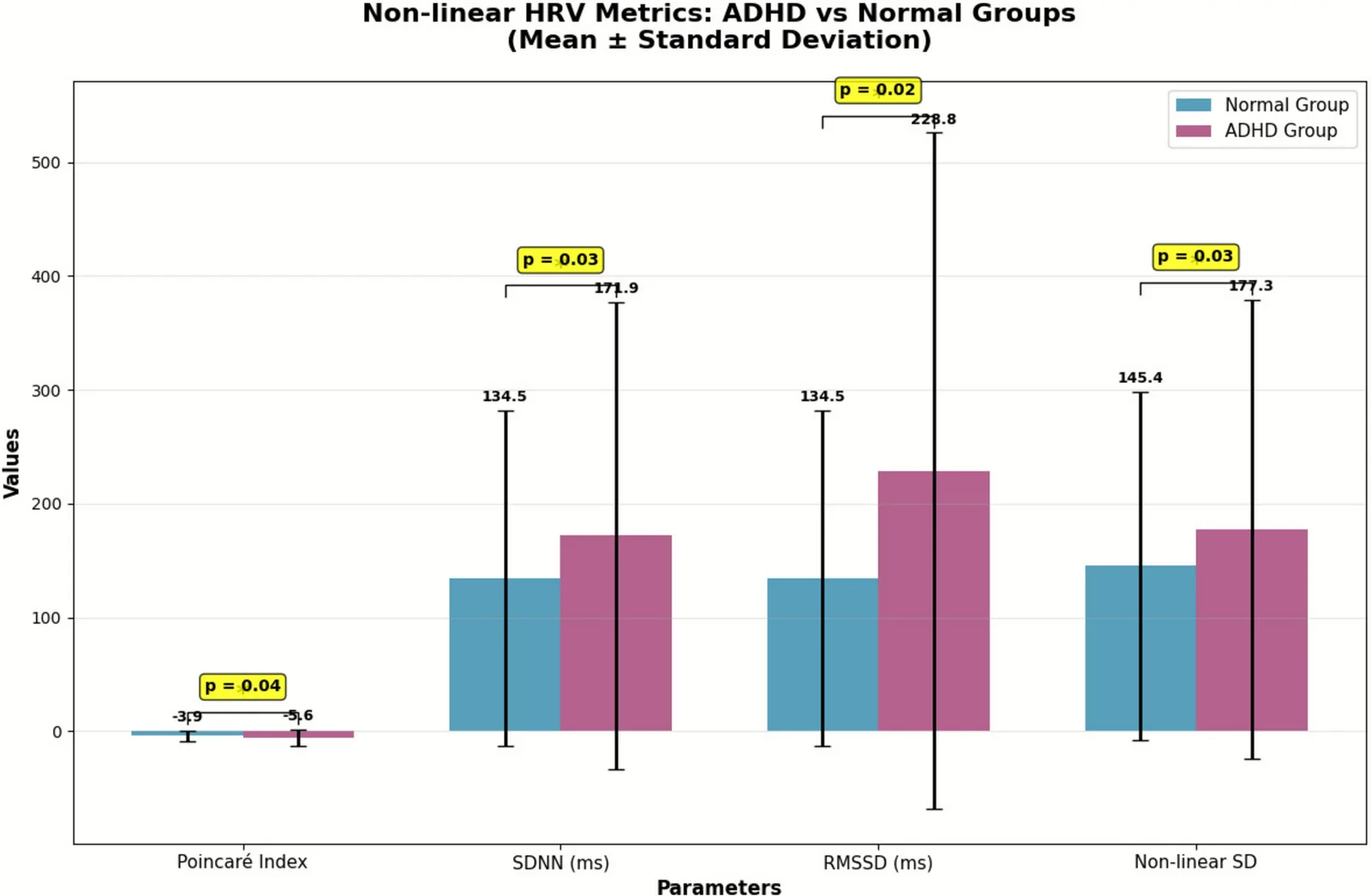
The non-linear heart rate variability analysis reveals significant elevations across all measured parameters in ADHD participants compared to normal controls. These consistent increases in Poincaré index, SDNN, RMSSD, and non-linear SD indicate enhanced heart rate complexity and greater variability in the ADHD group. The findings demonstrate substantially altered cardiac autonomic regulation characterized by increased irregularity and complexity of heart rate dynamics. This pattern suggests a state of autonomic dysregulation in ADHD that may reflect the underlying physiological correlates of the disorder’s characteristic symptoms and behavioral manifestations

**Fig. 2:**
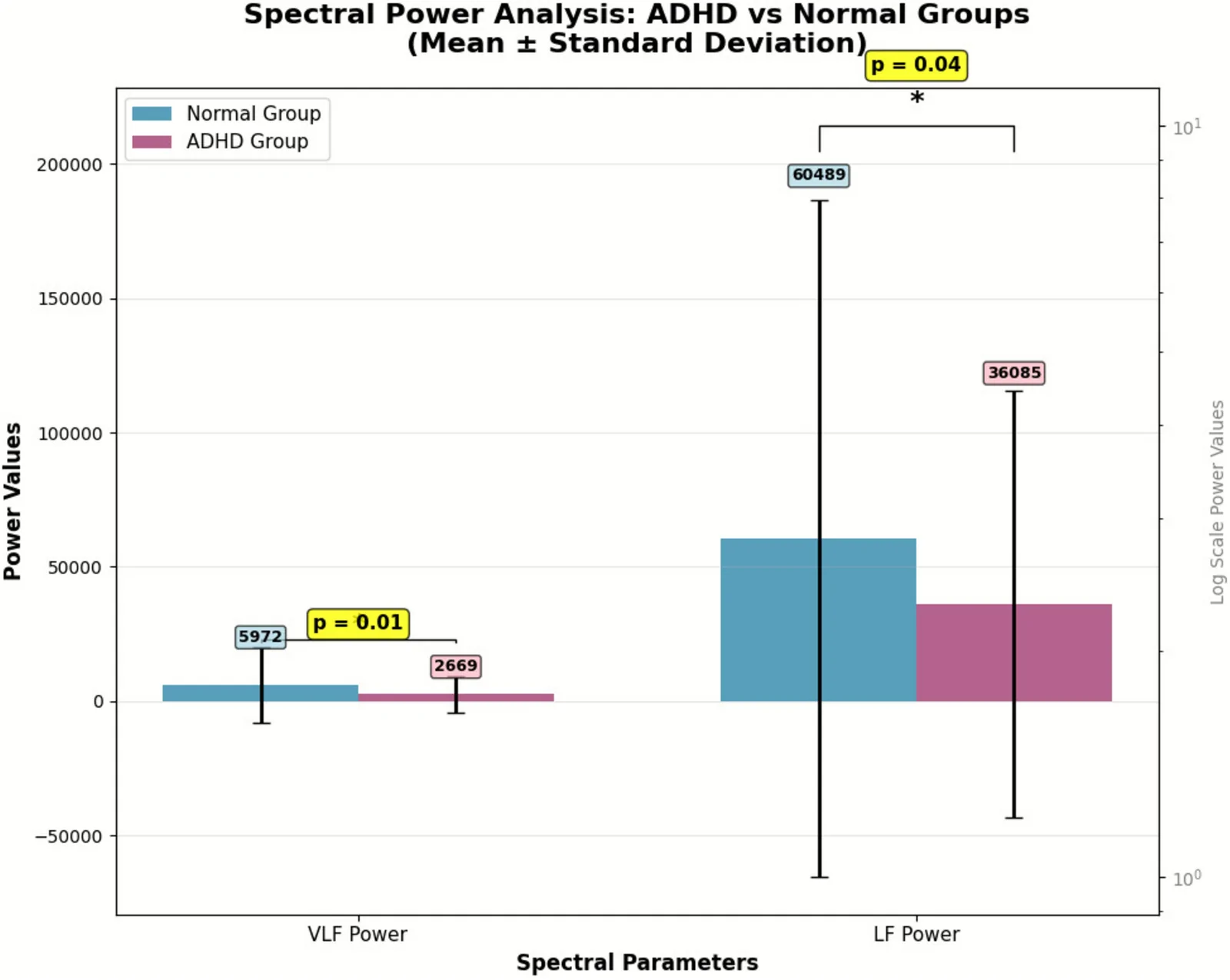
The spectral power analysis demonstrates significant reductions in both VLF and LF power components in ADHD participants compared to normal controls. These findings indicate substantial impairments in autonomic nervous system regulation among individuals with ADHD. The observed pattern of diminished spectral power reflects compromised parasympathetic activity and reduced autonomic flexibility, which may underlie the physiological mechanisms contributing to attention regulation difficulties characteristic of ADHD. The consistent suppression across multiple frequency bands suggests a broad disruption of cardiovascular regulatory systems in this population

The ADHD group exhibited slightly higher SDNN (171.88 ± 204.82 ms) and RMSSD values (228.82 ± 296.99 ms) compared with the Normal group (SDNN: 134.53 ± 147.30 ms; RMSSD: 177.35 ± 201.92 ms). These differences reached statistical significance in the unadjusted model (SDNN: *p* = 0.03; RMSSD: *p* = 0.02). However, ANCOVA models controlling for age and BMI were performed**.** After adjustment, the group differences for SDNN and RMSSD were no longer statistically significant (all *p* > 0.10). This suggests that the higher HRV values in the ADHD group may be partially explained by age- or BMI-related variance rather than group status alone. To address potential confounding from medication use, all analyses were repeated excluding medicated participants (n = 6). The pattern of results remained unchanged: SDNN and RMSSD did not differ significantly between groups (all *p* > 0.10). The direction of group means remained consistent with the full-sample analysis (Fig. 1, 2, Table 1, 2).

**Table 1:** According to the MOXO d-CPT results, differences in autonomic nervous system functioning between attention deficit hyperactivity disorder (ADHD) and normal children groups

Statistically significant result(s)	Comment on result
The ADHD group had a more negative Poincaré index of than the Normal group (*p* = 0.04)	A more negative Poincaré index in the ADHD group may indicate an imbalance, irregularity in the autonomic nervous system, specifically suggesting a weakened parasympathetic system and a more dominant sympathetic response
The standard deviation of NN intervals, SDNN, was higher in the ADHD group compared to the Normal group (*p* = 0.03)	Reflecting increased HR variability, DNN is a measure that reflects the influences of both the sympathetic and parasympathetic nervous systems. Therefore, the higher SDNN value in the ADHD group may suggest an alteration in autonomic nervous system balance
ADHD group showed higher RMSSD values (*p* = 0.02)	RMSSD is a sensitive marker of parasympathetic modulation. Higher RMSSD suggests that the ADHD group may exhibit increased parasympathetic activity
Power spectral analysis revealed that the Normal group had substantially higher VLF power (*p* = 0.01) and LF power (*p* = 0.04) than the ADHD group	Lower VLF in the ADHD group may indicate weaker autonomic adaptability and impaired central regulation of HRV
HF power was also greater in the Normal group compared to the ADHD group (*p* = 0.09)	Higher HF power is strongly associated with vagal (parasympathetic) modulation
ADHD group had a higher non-linear SD compared to the Normal group (*p* = 0.03)	Reflecting increased complexity in heart rate dynamics, ADHD group, reflecting greater complexity and unpredictability in heart rate patterns. Increased non-linear SD could also be a sign of impaired autonomic regulation in ADHD
ApEn values were slightly higher in the normal group compared to the ADHD group (*p* = 0.56)	Suggests a trend towards greater regularity and predictability in the heart rate patterns of the Normal group. The trend toward higher ApEn in the Normal group could imply that normal individuals experience greater autonomic adaptability and dynamic regulation

**Table 2:** Statistical results showing significant relationships between the MOXO d-CPT Attention performance, severity and heart rate variability (HRV) parameters, along with high effect sizes Cohen’s d in the independent sample t-test’s and *η*^2^ in the ANOVA

Attention	Significant statistical results	Effect size, Cohen d or *η*^2^
Performance	Despite variations in mean values across groups, the differences were not statistically significant	VLF: *η*^2^ = 0.080 (medium) Power VLF: *η*^2^ = 0.099 (medium) Total power VLF: *η*^2^ = 0.093 (medium) LF: *η*^2^ = 0.131 (medium) HF: *η*^2^ = 0.081 (medium) Power HF: *η*^2^ = 0.087 (medium) HRV Index: *η*^2^ = 0.128 (medium)
Severity	VLF: Extreme < Medium, *p* = 0.033 Total power VLF: Extreme < Medium, *p* = 0.029 LF: Extreme < Medium, *p* = 0.017	Min HR: d = 0.643 (moderate) Max HR: d = 0.910 (strong) Mean RR: d = 0.986 (medium-large) PNS index: d = -0.407 (moderate negative) SNS index: d = 0.484 (moderate) SDNN: d = 0.955 (large) RMSSD: d = 0.943 (large) VLF: d = 1.073 (strong) Power VLF: d = 0.498 (moderate)

The values in parentheses represent the degree of effect size. For HRV parameters abbreviations, please refer to the text

#### Heart Rate and RR Intervals

Mean heart rate was marginally higher in the ADHD group (97.06 ± 13.30 bpm) than in the Normal group (96.05 ± 13.13 bpm), but this difference was not statistically significant (*p* = 0.72). Minimum and maximum HR values also did not differ significantly (*p* = 0.43 and *p* = 0.15, respectively). Mean RR intervals were comparable between groups (*p* = 0.81).

#### Frequency-Domain Measures

Spectral analysis revealed significantly higher VLF (*p* = 0.01) and LF power (*p* = 0.04) in the Normal group compared to the ADHD group. HF power was also higher in the Normal group but only approached significance (*p* = 0.09). Since VLF and LF values displayed higher variance, ANCOVA analyses were also performed. Adjustment for age and BMI did not alter the pattern of group differences, and VLF remained significantly lower in the ADHD group.

#### Non-Linear Dynamics

The ADHD group showed a more negative Poincaré index ( − 5.61 ± 7.30) relative to the Normal group ( − 3.91 ± 4.65; *p* = 0.04), indicating increased signal irregularity. The non-linear SD was also significantly higher in the ADHD group (177.35 ± 201.92 vs. 145.43 ± 152.88; *p* = 0.03). Approximate entropy (ApEn) differences were not significant (*p* = 0.56). Although the ADHD group initially exhibited higher SDNN and RMSSD values, these effects did not remain significant after adjusting for age and BMI and were unaffected by medication-related sensitivity analyses. In contrast, VLF differences remained robust, consistent with autonomic dysregulation in ADHD (Fig. 1).

### Attention

#### Attention Performance

HRV and autonomic indices were compared across MOXO d-CPT attention performance subgroups (Good, Standard, Weak, Difficulty). Descriptively, the Good group showed higher mean HR (97.42 ± 13.91 bpm) and RR interval (629.04 ± 90.88 ms) compared to other groups, while the Weak group had the lowest mean HR (93.33 ± 13.72 bpm) and the most negative PNS index (–6.74 ± 10.95). SNS index values varied modestly across groups (Good: 2.14 ± 2.23; Weak: 1.11 ± 1.50). No differences reached statistical significance for physiological (mean, min, max HR, SDNN, RMSSD, stress index), frequency-domain (VLF, LF, HF, NN50, pNN50, RR triangular index, TINN), or non-linear measures (SD, non-linear SD, SD2/SD1, ApEn, SampEn; *p* = 0.082–0.906). Effect size analyses indicated moderate effects for LF (*η*^2^ = 0.131) and VLF (*η*^2^ = 0.080), while other measures, including Min HR (*η*^2^ = 0.044), Max HR (*η*^2^ = 0.053), and PNS/SNS indices (*η*^2^ = 0.023–0.024), showed minimal effects. *In summary, attention performance subgroups exhibited descriptive differences in HRV and autonomic measures, but only frequency-domain indices (LF, VLF) demonstrated moderate effect sizes, and no statistically significant group-level differences were observed*.

#### Attention Severity

Due to limited sub-severity cases, attention severity groups were merged: Low + Medium (“Medium,” n = 40) and High + Extreme (“Extreme,” n = 12). Most physiological and non-linear HRV measures—including mean, minimum, and maximum HR, SDNN, RMSSD, PNS/SNS indices, and SD2/SD1, ApEn, SampEn—showed no significant differences between groups, with small effect sizes (Cohen’s d = 0.05–0.35). Significant differences were observed in frequency-domain measures. The Extreme group exhibited lower VLF power, Total Power VLF, and LF power compared to the Medium group (Cohen’s d = 0.58–0.62), while HF power approached significance (Cohen’s d = 0.48). *Overall, apart from VLF, Total Power VLF, and LF power, HRV and autonomic measures did not differ significantly between merged severity groups, indicating largely similar autonomic regulation across attention severity levels, with reductions in frequency-domain indices associated with higher impairment* (Table 2).

### Timing

#### Timing Performance

Among attention performance groups, only VLF power differed significantly, with the Good performance group showing higher values than the Standard group (Cohen’s d = 0.42, *η*^2^ = 0.040). Total Power VLF and Power VLF log showed trends toward significance, while all other physiological (mean, min, max HR, SDNN, RMSSD, PNS/SNS indices) and non-linear measures (SD2/SD1, SampEn) exhibited no significant differences. Effect sizes for non-significant measures were generally small (*η*^2^ = 0.008–0.033), indicating modest group-level variation. Overall, apart from VLF power, HRV parameters showed no meaningful differences between performance groups.

#### Timing Severity

Attention severity subgroups were merged due to limited cases (Low + Medium: n = 7, High + Extreme: n = 16, Normal: n = 29). No significant differences were observed in mean HR, min HR, max HR, or mean RR (*p* = 0.173–0.870, *η*^2^ = 0.006–0.069). SDNN, RMSSD, NN50, pNN50, and PNS/SNS indices also did not differ significantly (*p* = 0.240–0.920, *η*^2^ = 0.008–0.057). In frequency-domain measures, only VLF power differed significantly (*p* = 0.025, *η*^2^ = 0.040), with the Good performance group showing higher values than Standard. Total Power VLF and Power VLF log approached significance (*p* = 0.067–0.085). Non-linear measures (SD2/SD1, SampEn, ApEn) showed no significant differences (*p* > 0.05, *η*^2^ ≤ 0.022). Effect sizes were mostly small to modest, with notable effects for Power VLF percent (*η*^2^ = 0.114), Max HR (*η*^2^ = 0.069), SNS Index (*η*^2^ = 0.057), and Stress Index (*η*^2^ = 0.060). *Overall, HRV and autonomic measures showed descriptive differences across severity levels, but group-based effects were generally non-significant, with only VLF power demonstrating a moderate significant difference* (Table 3).

**Table 3:** Statistical results showing significant relationships between the MOXO d-CPT timing performance, severity and heart rate variability (HRV) parameters, along with high effect sizes Cohen’s d in the independent sample t-test’s and *η*^2^ in the ANOVA

Timing	Significant statistical results	Effect size, Cohen d or *η*^2^
Performance	VLF Power: Good > Standard, *p* = 0.025	VLF: *η*^2^ = 0.176 (large) Power VLF log: *η*^2^ = 0.137 (large) Total power VLF: *η*^2^ = 0.128 (large)
Severity	Despite variations in mean values across groups, the differences were not statistically significant	Power VLF %: *η*^2^ = 0.114 (large) Power HF %: *η*^2^ = 0.057 (medium) Max HR (bpm): *η*^2^ = 0.069 (medium) SNS index: *η*^2^ = 0.057 (medium) Stress index: *η*^2^ = 0.060 (medium) NN50: *η*^2^ = 0.046 (medium) pNN50: *η*^2^ = 0.036 (small)

The values in parentheses represent the degree of effect size. For HRV parameters abbreviations, please refer to the text

### Impulsivity

#### Impulsivity Performance

Heart rate (HR) and related autonomic measures were assessed across MOXO d-CPT impulsivity groups: Good (N = 27), Standard (N = 15), Weak (N = 5), and Difficulty (N = 5). No statistically significant differences were found in mean, minimum, or maximum HR, RR intervals, or autonomic indices (PNS, SNS) between groups (*p* = 0.31–0.54). For example, mean HR ranged from 96.53 ± 16.34 bpm (Standard) to 99.11 ± 11.91 bpm (Good), and PNS Index ranged from − 3.68 ± 3.65 (Good) to − 9.45 ± 8.94 (Difficulty). Effect size analyses indicated moderate to large group-level differences for several HRV variables. Notably, VLF (*η*^2^ = 0.176), Power VLF log (*η*^2^ = 0.137), and Total Power VLF (*η*^2^ = 0.128) demonstrated the largest effects. Moderate effects were observed for SDNN (*η*^2^ = 0.079), RMSSD (*η*^2^ = 0.079), and NN50 (η^2^ = 0.102), whereas non-linear measures such as ApEn (*η*^2^ = 0.023) and Power LF (*η*^2^ = 0.012) showed small effects. *In summary, while HR and autonomic indices did not differ significantly across impulsivity performance groups, effect size analyses suggest meaningful variations in frequency-domain and some time-domain HRV measures* (Table 4).

**Table 4:** Statistical results showing significant relationships between the MOXO d-CPT Impulsivity performance, severity and heart rate variability (HRV) parameters, along with high effect sizes Cohen’s d in the independent sample t-test’s and *η*^2^ in the ANOVA

Impulsivity	Significant statistical results	Effect size, Cohen d or *η*^2^
Performance	Despite variations in mean values across groups, the differences were not statistically significant	VLF: *η*^2^ = 0.176 (large) Power VLF log: *η*^2^ = 0.137 (large) Total Power VLF: *η*^2^ = 0.128 (large) SDNN: *η*^2^ = 0.079 (moderate) RMSSD: *η*^2^ = 0.079 (moderate) NN50: *η*^2^ = 0.102 (moderate)
Severity	SDNN: Extreme > Normal, *p* = 0.043 RMSSD: Extreme > Normal, *p* = 0.034 SD: Extreme > Normal, *p* = 0.034 PNS Index: Extreme < Normal, *p* = 0.050	Mean HR: d = 0.386 (small) Min HR: d = 0.451 (medium) Max HR: d = -0.429 (small) Mean RR: d = -0.376 (small, negative) PNS Index: d = 0.907 (large) SNS Index: d = 0.369 (moderate) SDNN: d = -0.748 (large, negative) RMSSD: d = -0.796 (large, negative)

The values in parentheses represent the degree of effect size. For HRV parameters abbreviations, please refer to the text

#### Impulsivity Severity

Due to insufficient subgroups for analysis, participants were merged into two impulsivity severity groups: Extreme (High + Extreme, n = 38) and Normal (Low + Medium, n = 14). No significant differences were found in mean HR (92.00 ± 13.29 vs. 97.08 ± 13.17 bpm, *p* = 0.873), minimum HR (58.00 ± 33.46 vs. 70.21 ± 26.58 bpm, *p* = 0.356), or maximum HR (127.75 ± 13.28 vs. 121.06 ± 15.71 bpm, p = 0.793) between groups. Significant differences emerged in HRV measures. The Extreme group exhibited higher RMSSD (399.75 ± 428.39 ms vs. 191.32 ± 247.57 ms, *p* = 0.034), SDNN (284.78 ± 294.36 ms vs. 147.69 ± 173.85 ms, *p* = 0.043), and SD Value (283.28 ± 303.55 vs. 135.50 ± 175.39, *p* = 0.034), indicating greater variability. Additionally, the Extreme group had a lower PNS Index ( − 10.30 ± 10.10 vs.  − 4.55 ± 6.02, *p* = 0.050), reflecting reduced parasympathetic activity. No significant group differences were observed for NN50, pNN50, RR triangular index, TINN, Stress Index, or frequency-domain measures (VLF, LF, HF).

Effect size analyses revealed medium to large effects. Cohen’s d indicated small-to-medium effects for Mean HR (0.386) and Min HR (0.451), while the PNS Index showed a large effect (0.907). RMSSD ( − 0.796) and SDNN ( − 0.748) also demonstrated substantial effects, with higher impulsivity associated with greater HRV. Non-linear indices (SD, SampEn, ApEn) displayed smaller effects (0.3–0.5), suggesting more subtle group differences. *In summary, although basic HR measures did not differ, higher impulsivity severity was associated with increased HRV variability and reduced parasympathetic activity, with moderate-to-large effect sizes observed in key HRV indices.*

### Hyperactivity

#### Hyperactivity Performance

Participants were divided into two hyperactivity performance groups: Weak + Difficulty (n = 17) and Good + Standard (n = 35). No significant differences were observed in basic HR measures, including mean HR (98.34 ± 13.77 vs. 93.29 ± 11.26 bpm, *p* = 0.196), minimum HR (72.71 ± 28.09 vs. 62.18 ± 23.74 bpm, *p* = 0.189), or maximum HR (122.63 ± 17.16 vs. 119.41 ± 11.61 bpm, *p* = 0.489). The Weak + Difficulty group showed a higher mean RR interval (653.53 ± 84.79 vs. 622.14 ± 88.56 ms, *p* = 0.230).

In HRV indices, only the SNS Index reached statistical significance (*p* = 0.048), with the Good + Standard group exhibiting higher sympathetic activity, suggesting greater stress or arousal. Other measures, including PNS Index ( − 5.67 ± 7.80 vs. − 4.66 ± 5.80, *p* = 0.603), SDNN (141.97 ± 170.22 vs. 191.72 ± 214.53 ms, *p* = 0.369), RMSSD (185.81 ± 245.85 vs. 251.70 ± 304.85 ms, *p* = 0.406), NN50 (52.97 ± 44.10 vs. 68.76 ± 35.74, *p* = 0.205), and frequency-domain measures showed no significant differences. Non-linear measures, including ApEn and SampEn, also did not differ significantly.

Effect size analyses highlighted notable contrasts: moderate-to-large effects were observed for SNS Index (d = 0.491) and Stress Index (d = 0.489), while Mean HR (d = 0.388), Min HR (d = 0.394), and VLF B (d = 0.258) showed moderate effects. Other parameters, such as LF (d = 0.020), Total Power VLF ms^2^ (d = −0.066), and non-linear measures, displayed small or negligible effects. Negative moderate effects were found for Mean RR (d = −0.359) and NN50 (d = −0.380), and smaller negative effects for RMSSD (d = −0.248), SDNN (d = −0.268), and Power VLF log (d = −0.306).

*In summary, higher hyperactivity performance was associated with increased sympathetic activation (SNS Index), while other HRV and autonomic measures showed no significant group differences but moderate effect sizes for several variables, suggesting subtle physiological modulation related to hyperactivity levels* (Table 5).

**Table 5:** Statistical results showing significant relationships between the MOXO d-CPT Hyperactivity performance, severity and heart rate variability (HRV) parameters, along with high effect sizes Cohen’s d in the independent sample t-test’s and *η*^2^ in the ANOVA

Hyperactivity	Significant statistical results	Effect size, Cohen d or *η*^2^
Performance	SNS index, Good + Standard > Weak + Difficulty, *p* = 0.04	*Large effects* SNS index: d = 0.491 Stress index: d = 0.489 *Moderate effects* Mean HR: d = 0.388 Min HR: d = 0.394 VLF B: d = 0.258 *Small effects* LF: d = 0.020 Total power VLF: d = −0.066 * Negative moderate effects* Mean RR: d = −0.359 NN50: d = −0.380 Power VLF log: d = −0.306 RMSSD: d = −0.248 SDNN: d = −0.268 Power LF log: d = −0.299 Power HF log: d = −0.353 *Smaller negative effects* TINN: d = −0.132 RR triangular index: d = −0.478 Non-linear SD: d = −0.298
Severity	Despite variations in mean values across groups, the differences were not statistically significant	Min HR: d = 0.183 (small) Mean HR: d = 0.273 (moderate) SNS index: d = 0.387 (moderate) SD2/SD1 ratio: d = 0.393 (moderate) Stress index: d = 0.399 (moderate) VLF B: d = 0.347 (moderate)

The values in parentheses represent the degree of effect size. For HRV parameters abbreviations, please refer to the text

#### Hyperactivity Severity

In the comparison between the normal and extreme hyperactivity severity groups, the only significant difference observed was in the VLF, with a statistically significant finding (*p* = 0.025), and a moderate effect size (d = 0.347) for VLF B. Additionally, marginal differences were noted in Mean HR (*p* = 0.38) and Mean RR (p = 0.39), with small effect sizes of d = 0.273 and *d* = −0.268, respectively. The Stress Index (*p* = 0.20) and SNS Index (*p* = 0.22) showed moderate differences between the groups, with effect sizes of d = 0.399 and d = 0.387, respectively.

On the other hand, no significant differences were found for Min HR (*p* = 0.56), Max HR (*p* = 0.49), and PNS Index (*p* = 0.69). Other heart rate variability measures, including SDNN, RMSSD, NN50, pNN50, RR triangular index, and TINN, showed no statistically significant differences (all *p*-values > 0.20). Furthermore, the VLF A, Power VLF percent, Power VLF log, Power LF, Power LF log, Power HF, and various non-linear dynamics measures such as Non-linear SD, Non-linear ApEn, and SD Value also demonstrated no significant differences between the groups (all *p*-values > 0.20).

These findings suggest that, overall, there are only a few parameters that exhibit significant or marginal differences, while the majority of the measures show no statistical difference between the normal and extreme hyperactivity severity groups.

Findings from the HRV analysis indicate meaningful differences in autonomic nervous system (ANS) regulation between children with ADHD and typically developing controls. These results align with prior work suggesting that ADHD is characterized by altered sympathovagal balance and reduced autonomic stability (Clancy et al., 2013; Lázaro et al., 2015). The ADHD group exhibited more irregular autonomic patterns, as reflected by increased non-linear HRV complexity and altered Poincaré indices, consistent with models describing dysregulated neurovisceral integration in ADHD (Thayer & Lane, 2000, 2009).

The pattern of elevated SDNN and RMSSD values in some ADHD subgroups may reflect greater autonomic variability or compensatory parasympathetic adjustments under stress, as proposed in the neurovisceral integration model. However, these interpretations should be viewed cautiously, as the direction of HRV alterations in ADHD varies across studies and may depend on context, task demands, and symptom dimensions (Lázaro et al., 2015; Young et al., 2019). The control group demonstrated higher VLF and LF power, suggesting more organized and adaptable autonomic regulation. Lower VLF values in ADHD participants, particularly in lower-performing groups, are consistent with reduced adaptability to stressors and impaired self-regulatory capacity (Young et al., 2019). Although HF differences were nonsignificant, trends toward higher HF in the control group align with prior findings of attenuated parasympathetic activity in ADHD (Lázaro et al., 2015).

Notably, the strongest associations were observed in impulsivity and hyperactivity performance domains of the MOXO d-CPT. The Good impulsivity group displayed significantly lower VLF power than the Weak group, indicating more efficient autonomic regulation. This aligns with evidence linking parasympathetic stability to enhanced impulse control (O’Connell et al., 2014; Geisler et al., 2017). Higher RMSSD values in the Good impulsivity performers further support the role of vagal flexibility in behavioral regulation. In the impulsivity severity subgroup analysis, higher SDNN, RMSSD, and SD values in the Extreme group may reflect increased autonomic volatility rather than effective regulation, consistent with elevated emotional and behavioral lability often observed in high-impulsivity profiles.

Results in the hyperactivity domain were partly consistent with expectations. The Weak + Difficulty hyperactivity group demonstrated significantly higher SNS Index values, suggesting greater sympathetic activation linked to performance decrements. While HRV measures such as SDNN and RMSSD did not differ significantly, trends toward higher parasympathetic indices in the Good + Standard group may indicate more balanced autonomic modulation during task engagement (Thayer & Lane, 2000; O’Connell et al., 2014). Findings in the timing and attention domains were less pronounced, possibly due to greater heterogeneity in attentional deficits or lower sensitivity of HRV to sustained attention performance compared to impulsivity and motor-inhibition demands (Clancy et al., 2013).

Taken together, the findings support the neurovisceral integration model, suggesting that ANS flexibility is linked to prefrontal–subcortical network efficiency underlying cognitive control. The associations between autonomic parameters—particularly VLF and the SNS Index—and MOXO d-CPT performance indicate that HRV may serve as a physiological correlate of behavioral and cognitive control processes in ADHD.

Nevertheless, several limitations must be considered. The cross-sectional design precludes causal inferences, and the modest sample size, especially in severity subgroups, may have reduced power to detect smaller effects. The study relied on a single 5 min resting HRV recording, limiting evaluation of dynamic autonomic responses. Additionally, findings are specific to performance on the MOXO d-CPT and may not generalize to other cognitive domains or assessment tools.

## Conclusion

This study demonstrates that children with ADHD exhibit distinct autonomic profiles characterized by sympathovagal imbalance and irregular heart rate dynamics. Among the HRV parameters examined, VLF power and the SNS Index were most consistently associated with performance outcomes—particularly in impulsivity and hyperactivity domains of the MOXO d-CPT. These results support the neurovisceral integration model and highlight HRV as a promising objective biomarker for characterizing autonomic correlates of behavioral regulation in ADHD.

The combined use of comprehensive HRV profiling with the ecologically valid MOXO d-CPT represents a methodological strength, providing insight into physiological underpinnings of task performance under realistic distraction conditions. Future research should employ longitudinal designs with larger samples to clarify causal pathways, examine HRV patterns across ADHD subtypes, and evaluate HRV sensitivity to treatment effects, including neurofeedback, biofeedback, and pharmacological interventions.

## Study Limitations

The use of HRV measurements, which are frequently employed to evaluate ANS activity, is one of the study’s limitations. These measurements may not adequately capture the intricate dynamics of a person’s physiological and psychological state. Numerous factors, such as age, fitness level, medication, and emotional stress, can affect HRV and introduce variability that is not relevant to the intended research focus (Shaffer & Ginsberg, 2017). Therefore, even though HRV is a useful tool for assessing autonomic regulation, its interpretation needs to be done carefully because outside influences may have distorted the results, reducing their generalizability (Thayer & Lane, 2000; Task Force, 1996).

Additionally, the MOXO d-CPT has been criticized for its limited focus on specific aspects of attention, especially impulsivity, timing, and hyperactivity, despite its value in assessing cognitive performance (Barkley, 2015). Although it works well for identifying particular attention disorders, it does not fully address other areas of cognition like memory, executive function, and decision-making. The multifaceted nature of attention and cognitive control, which is known to involve intricate interactions between multiple cognitive and neural systems, may therefore not be adequately captured by the study’s findings, even though they are instructive in terms of attentional impairments (Posner & Petersen, 1990).

The sample size is another drawback, especially for subgroups that are categorized according to severity and performance. Our capacity to perform thorough subgroup analyses was hampered by the small sample sizes in these subgroups, which might have decreased the results’ statistical power and dependability (Button et al., 2013). This might have had an impact on our findings’ validity and generalizability. A larger sample size would have improved the study’s robustness and yielded more trustworthy information about the connections between attentional performance levels and autonomic regulation.

## Data Availability

The datasets generated during and/or analyzed during the current study are available from the corresponding author on reasonable request.
